# Next-Generation Targeted Molecular Therapies in Immune-Mediated Inflammatory Diseases

**DOI:** 10.34172/apb.46844

**Published:** 2026-05-10

**Authors:** Mostafa Akbarzadeh-Khiavi, Amir-Hossein Olfati, Azam Safary

**Affiliations:** ^1^Liver and Gastrointestinal Diseases Research Center, Tabriz University of Medical Sciences, Tabriz, Iran; ^2^Research Center for Pharmaceutical Nanotechnology, Biomedicine Institute, Tabriz University of Medical Sciences, Tabriz, Iran; ^3^Connective Tissue Diseases Research Center, Tabriz University of Medical Sciences, Tabriz, Iran

**Keywords:** Immune-mediated inflammatory diseases, Targeted molecular therapies, Precision medicine, Immune tolerance

## Abstract

Immune-mediated inflammatory diseases (IMIDs) are chronic disorders characterized by dysregulated immunity and loss of self-tolerance. Although significant progress has been made in the management of IMIDs, challenges such as drug resistance, immunogenicity, and limited accessibility continue to impede clinical outcomes. Achieving sustained remission necessitates a transition toward precision medicine that addresses fundamental disease mechanisms. This study examines recent advances in molecular interventions, including cytokine inhibitors, kinase modulators, and B-cell therapies, and emphasizes how these approaches improve remission rates and reduce relapses. Additionally, emerging platforms such as RNA therapeutics, nanomedicine, and chimeric antigen receptor (CAR) cell therapies are discussed, with a focus on their potential to enhance efficacy and prolong clinical response duration. Integrating these innovations with artificial intelligence to identify therapeutic targets, predict patient responses, and optimize drug development is essential to realizing truly personalized, more successful treatments for IMIDs.

## Introduction

 Immune-mediated inflammatory diseases (IMIDs), including rheumatoid arthritis (RA), systemic lupus erythematosus (SLE), ankylosing spondylitis (AS), and psoriatic arthritis (PsA), are chronic conditions characterized by dysregulated immune signaling and loss of self-tolerance. The activity of autoreactive immune cells leads to autoantibody production, proinflammatory cytokine release, tissue injury, organ dysfunction, and joint destruction, ultimately resulting in long-term disability and diminished quality of life.^1^

 Conventional therapies for IMIDs, including nonsteroidal anti-inflammatory drugs (NSAIDs), glucocorticoids, and synthetic disease-modifying antirheumatic drugs (DMARDs), function primarily through broad immunosuppression or immunomodulation. While these agents offer symptomatic relief, their use is constrained by significant adverse effects, such as increased infection risk, cytopenias, organ toxicity, and other complications. Importantly, these treatments do not address the fundamental immune intolerance underlying IMIDs.^2,3^

 Over the past two decades, significant advances in elucidating the molecular basis of immune dysregulation in IMIDs have driven the development of precision-targeted biological and small-molecule therapeutics. These innovations have transformed treatment paradigms by shifting the focus from symptomatic management to sustained clinical remission and the possibility of long-term drug-free remission. This review presents a focused analysis of current and emerging targeted molecular therapies, examining their mechanistic foundations, clinical efficacy, safety profiles, and limitations. Additionally, next-generation platforms are explored that may further redefine the therapeutic landscape of IMIDs.

## Current Targeted Molecular Therapies in IMIDs

###  Cytokine Inhibition: Targeting the Inflammatory Cascade

 Identifying key proinflammatory cytokines as central mediators of IMIDs enabled the development of biologic agents. Tumor necrosis factor-alpha (TNF-alpha) inhibitors, including adalimumab, etanercept, infliximab, golimumab, and certolizumab pegol, were the first successful targeted therapies. ^4^ These agents are highly effective for achieving clinical remission, inhibiting radiographic progression, and improving functional outcomes in RA, AS, and PsA, as demonstrated by randomized trials and long-term studies. ^5,6^ Their mechanism is neutralizing soluble and membrane-bound TNF-alpha. Etanercept acts as a decoy receptor, preventing TNF-alpha from binding its receptors and interrupting downstream inflammation. ^6,7^

 Following the success of TNF-alpha inhibition, blocking the interleukin-6 (IL-6) pathway emerged as a major therapeutic advance. In this context, tocilizumab, a humanized monoclonal antibody targeting the IL-6 receptor, is more effective than methotrexate as monotherapy for moderate-to-severe RA. ^8^ Moreover, tocilizumab can be combined with methotrexate for patients who respond poorly to DMARDs or TNF-alpha inhibitors. ^9,10^ Supporting its clinical utility, long-term extension studies confirm disease control, less joint damage, and sustained physical function for five years, with a stable and manageable safety profile. ^11-13^

 Further refinement in targeting the cytokine network has led to selective inhibition of the interleukin-17/interleukin-23 (IL-17/IL-23) axis, transforming the therapeutic landscape for PsA and AS. Secukinumab and ixekizumab, monoclonal antibodies against IL-17A, have demonstrated rapid and significant improvements in the signs and symptoms of AS and PsA in large phase III trials. ^14-16^ Secukinumab has shown particular efficacy in managing the axial manifestations of PsA, ^17^ while ixekizumab has also been shown to improve axial symptoms in patients with PsA. ^18^

 Expanding on these approaches, targeted therapies now include those directed at the upstream cytokine IL-23. Guselkumab, a monoclonal antibody that binds the p19 subunit of IL-23, has shown efficacy in phase III trials in biologic-naive patients with active PsA,^19^ and in patients previously treated with TNF-alpha inhibitors. ^20^ Guselkumab reduces downstream T helper 17 (Th17) effector cytokines, including IL-17A and IL-17F. This provides sustained efficacy in PsA by suppressing the pathway. ^21,22^ Long-term data show that guselkumab’s efficacy is maintained for at least 2 years. ^23^

###  Small-Molecule Kinase Inhibitors as Oral Precision Medicine

 The development of orally available small-molecule inhibitors represents a significant advancement in the management of IMIDs, providing an alternative to injectable biologic agents. These therapies are classified as Janus kinase (JAK) inhibitors and, more recently, as selective inhibitors of tyrosine kinase 2 (TYK2). JAK inhibitors, such as baricitinib, tofacitinib, upadacitinib, and filgotinib, enable broader inhibition of multiple cytokine signaling pathways, marking a paradigm shift in treatment strategies. ^24^ By targeting the JAK-STAT signaling cascade, these agents modulate responses from key inflammatory cytokines, including interleukins (ILs), interferons (IFNs), and growth factors. ^25,26^ In clinical trials, upadacitinib, a selective JAK1 inhibitor, produced higher clinical response and remission rates than the TNF inhibitor adalimumab in patients with RA who were unresponsive to methotrexate. ^27,28^ The five-year SELECT-COMPARE study confirmed that upadacitinib maintained higher response rates and remission over time, with a consistent safety profile. ^29,30^ Baricitinib, which inhibits JAK1 and JAK2, also showed sustained efficacy, with a manageable, unchanged long-term safety profile over a median of 4.6 years and up to 9.3 years. ^31,32^

 Recently, highly selective allosteric inhibition of TYK2 with deucravacitinib has emerged as a promising therapeutic approach. ^33^ Deucravacitinib binds to the TYK2 regulatory (JH2) domain, locking the enzyme in an inactive state. This selectivity for TYK2 over JAK1, JAK2, and JAK3 is associated with reduced adverse events commonly seen with less-selective JAK inhibitors, such as serious infections, thrombotic events, and hematologic issues. ^34,35^ In the phase 3 POETYK PSO-1 and PSO-2 trials, deucravacitinib achieved significantly higher efficacy than placebo and apremilast in moderate-to-severe plaque psoriasis, with sustained responses for up to three years. ^36,37^ In a phase 2 trial in active PsA, deucravacitinib led to greater improvements in the American College of Rheumatology-20 (ACR-20) response and other secondary measures compared with placebo. ^38^

###  B-Cell-Directed Therapies for Humoral Autoimmunity

 B lymphocytes play a central role in the pathogenesis of IMIDs by producing autoantibodies, presenting antigens, activating T cells, and secreting proinflammatory cytokines. Targeting B cells is a key therapeutic strategy for these conditions. Rituximab, a chimeric monoclonal antibody (mAb) directed against the CD20 antigen on B cells, induces profound and sustained B-cell depletion. ^39^ Foundational trials demonstrated its efficacy in combination with methotrexate for patients with RA, ^40^ and it is also used in severe, refractory SLE. ^41^ Although large randomized trials in SLE did not achieve their primary endpoints, multiple observational studies and meta-analyses indicate that rituximab may be effective for refractory lupus, including improved outcomes in lupus nephritis and reduced steroid requirements. ^42,43^ Incomplete B-cell depletion in some patients may contribute to suboptimal clinical responses in RA and SLE. ^44^ The safety profile is generally acceptable, although infusion-related reactions and rare serious adverse events, such as progressive multifocal leukoencephalopathy, have been reported with long-term use. ^45^

 Belimumab, a fully human mAb, provides an alternative B-cell-targeted therapy by neutralizing B-cell activating factor (BAFF), also known as B-lymphocyte stimulator (BLyS). BAFF is essential for B cell survival, differentiation, and class-switch recombination. ^46^ By blocking BAFF, belimumab disrupts the formation of highly active BAFF 60-mers and impairs B-cell maturation, thereby reducing autoantibody production. ^47^ Large randomized controlled trials have confirmed the efficacy and safety of belimumab in active SLE, supporting its regulatory approval. ^48,49^ The BLISS-LN trial further demonstrated that the addition of belimumab to standard therapy significantly improves renal outcomes over 2 years in patients with active lupus nephritis, reducing the risk of renal-related events or death. ^50^ The safety profile remains favorable, with no significant increase in adverse events compared to placebo. ^51^

###  Modulation of T-Cell Costimulation and Immune Checkpoints

 Abatacept is a soluble fusion protein that combines the extracellular domain of cytotoxic T-lymphocyte antigen-4 (CTLA-4) with a modified Fc portion of human IgG1. It selectively modulates T-cell activation by blocking the critical costimulatory pathway involving CD28 on T cells and its ligands, CD80 and CD86, on antigen-presenting cells. ^52,53^ Without this second signal, T-cell activation is attenuated, leading to a downstream reduction in cytokine production such as IL-6, inhibition of B-cell autoantibody production, and reduced activation of other inflammatory cells. ^54^ Abatacept has proven effective for disease control in RA patients, including those with an inadequate response to methotrexate or TNF inhibitors. ^55,56^ Recent evidence also suggests that abatacept can downregulate FcγRI expression on monocytes, potentially suppressing inflammatory responses mediated by immune complexes. ^57^ It generally has a favorable safety profile, with a lower risk of serious infections than some other biologic agents. ^54^

 In addition to established therapies, recent research underscores the therapeutic potential of modulating alternative immune checkpoint pathways. The programmed cell death protein 1 (PD-1) pathway serves as a critical immune checkpoint that maintains self-tolerance by inhibiting T cell activity. ^58,59^ Dysregulation of this pathway is associated with several autoimmune diseases. ^60^ Although PD-1 blockade with checkpoint inhibitors has transformed cancer treatment, it may also induce or worsen autoimmune conditions. ^61^ Conversely, strategies to activate the PD-1 pathway are being investigated as therapies for autoimmunity. For example, an experimental immunotoxin targeting PD-1-expressing cells ameliorated disease in mouse models of type 1 diabetes and experimental autoimmune encephalomyelitis, while preserving normal immune function. ^62^ These findings indicate that selective targeting of autoreactive T cells via the PD-1 pathway may provide a promising strategy for modulating immune responses in autoimmune diseases. ^63^

## Next-Generation Therapeutic Platforms for IMIDs

 While established molecular therapies have improved clinical outcomes, some IMIDs patients still experience treatment failure or adverse events, highlighting the need for next-generation therapeutic platforms. ^64^ To address these gaps, new approaches are emerging and include (i) chimeric antigen receptor (CAR) cell therapies; ^65,66^ (ii) RNA therapeutics, such as small interfering RNA (siRNA) technologies that selectively silence disease-associated genes; ^67^ (iii) nanotechnology-based targeted delivery systems for selective, inflammation-triggered drug administration; ^37,68^ and (iv) autologous hematopoietic stem cell transplantation (aHSCT), which seeks to reset the immune system and achieve sustained, drug-free remission in severe, refractory cases. ^69^ An overview of the major therapeutic classes currently used or under investigation in IMIDs is summarized in Table 1. Advances in precision immunology and bioengineering may transform rheumatologic care by shifting the focus from symptomatic relief to deep disease modification and, for some patients, a functional cure.

**Table 1:** Overview of Key Therapeutic Classes in IMIDs.

**Therapy Class**	**Examples**	**Targets**	**Diseases **	**Advantages**	**Limitations**	**References**
Cytokine Inhibitors	Adalimumab, Tocilizumab	TNF-alpha, IL-6 receptor	RA, AS	Rapid symptom relief, established efficacy	Infection risk, injection-site reactions	^70,71^
Kinase Inhibitors	Baricitinib, Upadacitinib	JAK/STAT pathway	RA, PsA	Oral administration, broad cytokine suppression	Thrombotic events, laboratory monitoring required	^31,72^
Cell-Targeted	Rituximab, CD19 CAR-T Cells	B cells, autoreactive lymphocytes	SLE, RA	Sustained remission, immune reset	High costs, infusion-related risks	^3,41^
RNA/Nano-Based	siRNA liposomes, mRNA Tregs	Gene expression, inflamed tissues	SLE, RA	High precision, tolerance induction	Developmental stage, delivery hurdles	^73,74^

* TNF-alpha: Tumor necrosis factor alpha; IL-6: Interleukin-6; JAK/STAT: Janus kinase/Signal transducer and activator of transcription; TYK2: Tyrosine kinase 2; BAFF: B-cell activating factor; CAR: Chimeric antigen receptor; CRS: Cytokine release syndrome; siRNA: Small interfering RNA; mRNA: Messenger RNA; Treg: Regulatory T cell; lncRNA: Long non-coding RNA; RA: Rheumatoid arthritis; AS: Ankylosing spondylitis; PsA: Psoriatic arthritis; SLE: Systemic lupus erythematosus.

###  Chimeric Antigen Receptor (CAR) Cell Therapies

 CAR T-cell therapy represents a novel immunotherapeutic strategy originally developed in oncology and now applied to the treatment of severe, refractory IMIDs. ^75^ This approach involves the genetic modification of T cells, sourced either from patients (autologous) or healthy donors (allogeneic), to express synthetic chimeric antigen receptors (CARs). These engineered receptors specifically recognize and bind to antigens on the surface of target cells, facilitating their destruction independently of the major histocompatibility complex (MHC). ^66^ CD19-targeted CAR T-cell therapy has demonstrated remarkable efficacy in small cohorts of patients with severe, refractory SLE. ^76^ Case series have shown that a single infusion of CD19 CAR T-cells induces profound B-cell aplasia, eliminates autoantibodies, and results in drug-free remission in all treated individuals. ^77,78^ In these reports, disease activity scores reached zero within three months, and remissions persisted for over two years in some cases, significantly surpassing outcomes achieved with conventional therapies. ^78,79^

 Allogeneic, or “off-the-shelf,” CAR cell products derived from healthy donors present several advantages over autologous approaches. ^80^ These products eliminate the need for individualized manufacturing, reduce associated costs, and mitigate production failures that may arise when using T cells from heavily pretreated patients. ^80,81^ Recent pilot studies of allogeneic anti-CD19 CAR T-cells in refractory SLE have demonstrated clinical remission rates comparable to those observed with autologous products. ^81,82^ Notably, these studies have reported favorable safety profiles, with only mild cytokine release syndrome (CRS) and no cases of graft-versus-host disease (GvHD), a significant concern in allogeneic therapies. ^81,83^

 CAR-engineered natural killer (NK) cells are being investigated as an alternative to T cells. These cells may provide safety advantages and can be produced from allogeneic donors for off-the-shelf application. ^84^ A phase 1 clinical trial is currently assessing an allogeneic anti-CD19 CAR-NK product (NKX019) in patients with refractory SLE. ^85^

 Despite its notable clinical efficacy, several significant barriers restrict the broader implementation of CAR-T cell therapy in IMIDs. These challenges include high manufacturing costs and complex logistics, the risk of toxicities and severe adverse effects such as CRS and immune effector cell-associated neurotoxicity syndrome (ICANS), and long-term risks related to sustained B-cell aplasia, including increased susceptibility to infections and potential secondary malignancies. ^80,86-88^

 Current research aims to address these limitations by developing strategies such as dual-targeting CARs, for example, those targeting both CD19 and BCMA, to prevent relapse due to antigen escape. This approach has demonstrated success in achieving sustained remission in refractory SLE. ^89,90^ In addition to SLE, CAR T-cell therapy is under investigation for other refractory IMIDs, with case series and pilot studies reported in systemic sclerosis (SSc), ^78,91^ idiopathic inflammatory myopathies (IIM), including antisynthetase syndrome, ^78,92^ and ANCA-associated vasculitis (AAV). ^93,94^ These findings indicate that the profound immune “reset” induced by this therapy may have broad applicability across antibody-mediated autoimmune diseases. ^95^ Another emerging strategy involves engineering regulatory T cells (Tregs) to express CARs. Instead of depleting immune cells, CAR-Tregs are engineered to migrate to sites of inflammation and actively suppress autoimmune responses, which promotes immune tolerance. ^96^ This method combines the antigen specificity of CARs with the inherent immunosuppressive properties of Tregs and has shown promise in preclinical models of colitis and other autoimmune disorders. ^97^

###  RNA Therapeutics: Precision Gene Silencing in IMIDs

 RNA-based therapeutics, such as siRNA, antisense oligonucleotides (ASOs), and messenger RNA (mRNA), facilitate highly specific, sequence-directed modulation of disease-associated genes, thereby providing exceptional precision and reducing off-target effects. ^98,99^ This approach allows for intervention at the post-transcriptional level and represents a novel strategy for the management of IMIDs. ^100^

 Long non-coding RNAs (lncRNAs) have been identified as essential regulators of immune homeostasis and are frequently dysregulated in IMIDs. Molecules such as NEAT1, GAS5, and HOTAIR modulate key inflammatory signaling pathways, including IL-6/STAT3 and TNF-alpha/NF-κB, and influence the differentiation of T and B cells. ^101-103^ Expression profiles of lncRNAs often correlate with disease activity in RA and SLE, suggesting their potential as biomarkers and therapeutic targets. ^104,105^ For instance, upregulation of HOTAIR and THRIL has been observed in patients with RA and was associated with elevated TNF-alpha levels, while altered GAS5 levels have been reported in both SLE and RA. ^105^ Preclinical studies have demonstrated the therapeutic potential of targeting these pathogenic molecules. A cationic liposome formulation has been developed for systemic delivery of siRNA to silence the proinflammatory cytokine TNF-alpha in experimental arthritis models. ^106^ Lipid nanoparticles designed for minimal burst release of TNF-alpha siRNA have demonstrated significant activity against RA, even in methotrexate-unresponsive models, by reducing bone loss and improving histopathological scores. ^107^

 Beyond silencing pathogenic genes, mRNA lipid nanoparticle platforms are being developed to engineer immune cells for adoptive cell therapy. This strategy enables the generation of immunosuppressive Tregs by delivering mRNA encoding key transcription factors, such as Foxp3, and antigen-specific T-cell receptors. ^73^ These ex vivo engineered Tregs show enhanced suppressive function and can be directed to specific tissues. ^73,108^ Compared to viral transduction, this method reduces the risk of insertional mutagenesis due to the transient nature of mRNA expression and allows for faster manufacturing. ^109^ In addition to engineering immune cells, another approach uses non-inflammatory mRNA-LNP constructs encoding disease-relevant autoantigens to induce antigen-specific tolerance. In mouse models of multiple sclerosis (MS), this method suppressed disease by expanding antigen-specific Tregs, highlighting its potential to restore self-tolerance without systemic immunosuppression. ^110,111^ Despite these advances, the clinical translation of RNA therapeutics remains challenging. Key issues include optimizing delivery vehicles for targeted biodistribution, protecting RNA from nuclease degradation, ensuring efficient cellular uptake and endosomal escape, and confirming long-term safety by minimizing off-target effects and unwanted immune activation. ^112,113^

###  Nanotechnology-Based Targeted Delivery Systems in IMIDs

 Nanotechnology platforms include biodegradable polymeric nanoparticles, liposomes, micelles, dendrimers, and stimuli-responsive hydrogels. These advanced systems provide targeted drug delivery to inflammatory sites, which reduces systemic exposure and toxicity. ^68,114^ They can be designed to release drugs in response to specific features of inflamed tissues, including acidic pH, high levels of reactive oxygen species (ROS), unique enzyme activities such as matrix metalloproteinases and cathepsins, or increased temperature. ^115,116^

 For example, folate receptor-targeted, pH-responsive nanocarriers encapsulating methotrexate or glucocorticoids have demonstrated increased therapeutic efficacy and reduced systemic adverse effects in preclinical models of arthritis. ^117^ The folate receptor, which is overexpressed on activated macrophages within inflamed synovium, facilitates selective nanoparticle uptake through receptor-mediated endocytosis. ^118^ In adjuvant-induced arthritis rat models, folate-conjugated polymeric nanoparticles containing methotrexate resulted in greater drug accumulation in arthritic joints compared to healthy joints, leading to improved arthritis score reduction and cartilage preservation relative to free methotrexate at equivalent doses. ^119^ Similarly, folate-targeted liposomes delivering dexamethasone, combined with ultrasound-targeted microbubble destruction, have enhanced drug release and significantly decreased joint swelling and inflammation in RA models. ^120^ In addition, nanoparticles responsive to ROS and loaded with anti-inflammatory agents also enable triggered drug release specifically within oxidatively stressed arthritic joints ^121-123^. For instance, celastrol-loaded bilirubin nanoparticles target ROS at pathological sites, thereby improving anti-arthritic efficacy and reducing toxicity in RA models. ^124^ Furthermore, pH-responsive multifunctional manganese dioxide nanoparticles enhance methotrexate delivery. They ameliorate the RA microenvironment by eliminating ROS and generating oxygen, thereby regulating inflammation and promoting macrophage polarization. ^125^

 Advanced multifunctional theranostic nanomedicine facilitates real-time monitoring of drug biodistribution, therapeutic efficacy, and disease progression through non-invasive imaging modalities, including magnetic resonance imaging (MRI), positron emission tomography (PET), and optical imaging. ^126,127^ For instance, ceria-based nanotheranostic agents for RA combine ROS scavenging with imaging capabilities. ^128^ Additionally, a theranostic nanoprobe that integrates activatable near-infrared (NIR-II) fluorescence imaging with synergistic immunotherapy, by releasing carbon monoxide and inhibiting IL-6 signaling, enables precise diagnosis and treatment of rheumatoid arthritis. ^129^

 Tolerogenic nanoparticles conjugated with disease-relevant autoantigens and immunomodulatory molecules can induce antigen-specific immune tolerance by promoting Tregs expansion and DCs tolerization without causing broad immunosuppression. ^130^ For example, lignin-based tolerogenic nanoparticles loaded with autoantigens have demonstrated the capacity to scavenge ROS and induce robust antigen-specific immune tolerance through Treg induction in experimental autoimmune encephalomyelitis (EAE) models. ^131^ Similarly, biomaterial-based therapeutic vaccines that carry self-antigens and tolerance-inducing inorganic nanoparticles, such as cerium oxide nanoparticles, can suppress activation of antigen-presenting cells (APCs). This approach enhances antigen-specific immune tolerance, resulting in recovery in EAE models. ^132^

 Recent advancements include biomimetic nanoparticles coated with Treg cell membranes, which retain native targeting ligands and immune-evasive characteristics. ^3^ Nanoparticles coated with Treg membranes and loaded with immunosuppressive agents exhibit extended circulation times and preferentially accumulate in inflamed lymph nodes in experimental autoimmune encephalomyelitis models, resulting in improved therapeutic efficacy compared to uncoated counterparts. ^133,134^ Furthermore, self-assembled peptide-based nanofibers displaying multiple autoantigenic epitopes can interact with B-cell receptors on autoreactive B cells, leading to anergy or apoptosis without inducing systemic immunosuppression. ^135^ These peptide-based self-assembling systems are under investigation as vaccine platforms and for oral immunization, offering controlled epitope presentation and enhanced stability. ^136-138^

###  Autologous Hematopoietic Stem Cell Transplantation (aHSCT) for IMIDs

 The aHSCT constitutes an intensive immunoablative strategy that induces a profound reset of the immune system, facilitating long-term drug-free remission in certain patients with severe, refractory IMIDs. ^139^ The procedure involves administration of high-dose immunosuppressive or myeloablative conditioning regimens, followed by reinfusion of previously collected autologous CD34 + hematopoietic stem cells. ^139^ The conditioning phase aims to eliminate pathogenic immune cell clones. Subsequent immune reconstitution from stem cells restores self-tolerance by promoting thymic re-education and diversification of the T-cell receptor repertoire. ^140^ Regarding immune cell recovery, evidence indicates that B-cell numbers recover within 3 months post-transplantation and may exceed baseline levels after 1 year, resulting in a predominantly naive immune phenotype. However, memory B cell populations recover more slowly, remaining below normal levels and exhibiting reduced repertoire diversity for up to a year. ^140^ In clinical practice, aHSCT is increasingly employed in aggressive MS and, compared to immune-reconstitution therapies such as cladribine and alemtuzumab, has demonstrated greater efficacy in suppressing relapses and promoting neurological function recovery in patients with active relapsing-remitting MS and moderate disability. ^139^ The therapeutic benefit of aHSCT likely results from a complex interplay between immune suppression and subsequent immune reconstitution. ^139^ Furthermore, beyond MS, aHSCT is also considered for patients with AL amyloidosis who are unresponsive to induction therapy; these patients may benefit from high-dose chemotherapy and aHSCT, with significant responses observed in those achieving a very good partial response or better following transplantation. ^141^ Despite these therapeutic advantages, aHSCT carries a substantial risk of treatment-related mortality, necessitating careful patient selection and specialized transplantation expertise. Notably, delayed complications, primarily infections, have been reported in 34% of MS patients treated with aHSCT in one study, although no treatment-associated deaths occurred in that cohort. ^139^

## Future Directions, Challenges, and Barriers to Clinical Implementation

 Despite remarkable progress, considerable challenges persist in the clinical implementation of therapeutics for IMIDs. High costs associated with biologics and advanced therapies restrict accessibility, especially in low- and middle-income countries, thereby exacerbating global health disparities. ^66,142^ For instance, advanced therapies such as CAR T-cell therapy, despite their promise for IMIDs, present significant financial obstacles. ^143^

 The development of anti-drug antibodies against biologics represents another significant challenge, as it can lead to secondary treatment failure. ^144^ While biosimilars have shown immunogenicity profiles similar to their reference biologics, immune responses to a range of immunomodulatory agents continue to influence treatment outcomes. ^66,145^

 Inter-individual molecular heterogeneity, influenced by genetic background, microbiome composition, and environmental exposures, as well as pathway redundancy, results in variable treatment responses among patients with similar clinical disease phenotypes. ^146,147^ This variability underscores the need for more precise treatment strategies. Addressing these challenges requires biomarker-driven clinical trials that utilize predictive enrichment strategies to identify patient subgroups most likely to benefit from specific interventions. ^148^ The introduction of biosimilar and generic versions of established biologics can enhance affordability and access, as studies have shown substantial cost savings without compromising efficacy or safety. ^66^ Global collaboration through international consortia, harmonized clinical trial protocols, and equitable distribution mechanisms, together with health policy initiatives such as value-based pricing models and expanded insurance coverage, is crucial for ensuring equitable access to these therapies worldwide. ^142^

 The future of IMID therapeutics is advancing toward multimodal precision medicine approaches that integrate next-generation platforms with advanced computational methodologies. Artificial intelligence (AI) and machine learning (ML) algorithms, when applied to multi-omics datasets such as genomics, transcriptomics, proteomics, and metabolomics, accelerate target identification, biomarker discovery, and patient stratification. These AI-driven approaches facilitate a deeper mechanistic understanding of disease processes and enable the prediction of drug safety and efficacy. ^149,150^

 AI is also used to optimize drug and dose parameters in combinatorial nanomedicine, addressing the time-, dose-, and patient-specific aspects of drug synergy. ^151^ In addition, AI-optimized CAR designs that incorporate predictive models of antigen binding affinity, T-cell activation kinetics, and toxicity risk scores, together with CRISPR-based gene-editing strategies to enhance CAR T-cell persistence and reduce exhaustion, have the potential to improve the efficacy, safety, and durability of cellular therapies. ^152-155^ However, challenges such as off-target effects, immune responses, and delivery methods must still be addressed to further improve the efficacy and safety of CRISPR-based therapies.

## Conclusion

 Targeted molecular therapies have transformed IMID management by shifting from broad immunosuppression to precise immunomodulation. Biologics and small-molecule inhibitors have improved disease control, clinical outcomes, and patient quality of life. CAR T-cell therapy, RNA therapeutics, nanotechnology-based drug delivery, and aHSCT offer the potential for sustained remission and, in some cases, functional cures. Advances in precision immunology, bioengineering, and AI are creating personalized, mechanism-based treatments that address the root immunological causes. AI and machine learning with multi-omics data accelerate target identification, biomarker discovery, patient stratification, and drug repurposing, thereby reducing development timelines and costs. High costs, immunogenicity, and variable treatment responses due to molecular heterogeneity remain major barriers. Continued translational research, robust clinical validation, and global collaboration are essential to overcome these challenges.

## Competing interests

 The authors declare that they have no competing interests.

## Ethical Approval

 The study was approved by the Ethics Committee of Tabriz University of Medical Sciences (IR.TBZMED.FMD.REC.1404.485).
